# Feasibility study of a clinically-integrated randomized trial of modifications to radical prostatectomy

**DOI:** 10.1186/1745-6215-13-23

**Published:** 2012-02-24

**Authors:** Andrew J Vickers, Caroline Bennette, Karim Touijer, Jonathan Coleman, Vincent Laudone, Brett Carver, James A Eastham, Peter T Scardino

**Affiliations:** 1 Department of Epidemiology and Biostatistics, Memorial Sloan-Kettering Cancer Center, 1275 York Avenue, New York, New York 11215 USA; 2 Department of Pharmacy, University of Washington, Seattle, Washington 98195, USA; 3 Department of Surgery, Memorial Sloan-Kettering Cancer Center, 1275 York Avenue, New York, New York 11215 USA

**Keywords:** Randomized controlled trials, surgery, research design, prostate cancer

## Abstract

**Abstract:**

**Background**

Numerous technical modifications to radical prostatectomy have been proposed. Such modifications are likely to lead to only slight improvements in outcomes. Although small differences would be worthwhile, an appropriately powered randomized trial would need to be very large, and thus of doubtful feasibility given the expense, complexity and regulatory burden of contemporary clinical trials. We have proposed a novel methodology, the clinically-integrated randomized trial, which dramatically streamlines trial procedures in order to reduce the marginal cost of an additional patient towards zero. We aimed to determine the feasibility of implementing such a trial for radical prostatectomy.

**Methods**

Patients undergoing radical prostatectomy as initial treatment for prostate cancer were randomized in a factorial design to involvement of the fascia during placement of the anastomotic sutures, urethral irrigation, both or neither. Endpoint data were obtained from routine clinical documentation. Accrual and compliance rates were monitored to determine the feasibility of the trial.

**Results**

From a total of 260 eligible patients, 154 (59%) consented; 56 patients declined to participate, 20 were not approached on recommendation of the treating surgeon, and 30 were not approached for logistical reasons. Although recording by surgeons of the procedure used was incomplete (~80%), compliance with randomization was excellent when it was recorded, with only 6% of procedures inconsistent with allocation. Outcomes data was received from 71% of patients at one year. This improved to 83% as the trial progressed.

**Conclusions**

A clinically-integrated randomized trial was conducted at low cost, with excellent accrual, and acceptable compliance with treatment allocation and outcomes reporting. This demonstrates the feasibility of the methodology. Improved methods to ensure documentation of surgical procedures would be required before wider implementation.

**Trial registration:**

ClinicalTrials.gov NCT00928850

## Background

A radical prostatectomy (RP) is one of the most complex operations performed by urologists. The ideal outcome of a RP is complete removal of the cancer with no sunsequent recurrence, minimal blood loss, no serious perioperative complications and complete recovery of erectile and urinary function. No surgeon achieves such results uniformly and it appears likely that results are highly sensitive to fine details of surgical technique.

Considerable recent data suggests that outcomes of RP are indeed surgeon dependent. The largest body of evidence comes from what are known as "volume outcome" studies: typically, an administrative database, such as Medicare, is searched and surgical outcomes recorded along with details of the number of procedures conducted by a surgeon in the previous 12 months. It has been reported that surgeons with a higher yearly caseload have better outcomes [1-3] and that there is greater than chance variation between surgeons even adjusting for volume [1,2,4].

It seems reasonable to suppose that, if the results of RP differ by surgeon, there must be identifiable aspects of surgical technique that affect outcome. The urologic literature is replete with modifications to the contemporary standard RP. As some illustrative examples, investigators have recommended modifications such as avoidance of countertraction during nerve sparing [5]; transverse camera port incision for robotically-assisted RP [6]; lateral view dissection of the prostato-urethral junction during laparoscopic RP [7]; posterior reconstruction of the rhabdosphincter [8]; division of the dorsal venous complex before rather than after suture ligation [9].

We have previously argued that the increasing cost, complexity and regulatory burden of contemporary clinical trials makes it infeasible that these sorts of modifications could be subject to randomized comparison [10]. For example, imagine that avoidance of countertraction during nerve sparing could improve rates of erectile function from 50% to 55%, a small but highly worthwhile benefit given that it results from a minor surgical modification. A trial with sufficient power detect this effect size would require more than 3000 patients. Given that a typical randomized trial costs over $5000 per patient [11], the trial budget might approach $20 million. It is highly unlikely that any funder would consider such a trial.

We have proposed a novel methodology - the clinically-integrated randomized trial - in an effort to dramatically decrease trial costs and therefore enlarge the number of clinical questions that can be addressed by randomization. The key aspect of our methodology is that the clinical experience of the patient and doctor is virtually indistinguishable whether or not the patient is randomized, primarily because trial endpoints are obtained from routine clinical data. Trial patients go through informed consent procedures, and certain aspects of care, such as modifications to the surgical technique used, are determined by randomization rather than being at the discretion of the doctor. Otherwise, there are no obvious differences between the clinical care, follow-up, payment and documentation requirements between patients who do and do not participate in the trial. The lack of trial specific procedures entails the marginal cost of putting an additional patient on trial becomes close to zero [10].

Here we report a feasibility study of clinically-integrated randomized trial of modifications to RP (NCT00928850). Our primary objective was to determine the acceptability of the trial to surgeons and patients, defined in terms of accrual rates and compliance.

## Methods

This was a single center, feasibility study of a randomized trial, approved by the Institutional Review Board of Memorial Sloan Kettering Cancer Center (MSKCC) in accordance with the Helsinki declaration (protocol number 09-051). The study took place in the urology clinics of MSKCC in New York, NY from July 2009 to April 2010. Eligible patients were those scheduled for radical prostatectomy for the treatment of prostate cancer with one of the consenting surgeons at MSKCC. Exclusion criteria was prior treatment for prostate cancer (radiation, hormonal therapy, chemotherapy or focal therapy).

Patients were randomized to irrigation, involvement of the fascia during placement of the anastomotic sutures, both or neither. A full description of each technique is given in additional file 1. In brief, the irrigation procedure involved irrigating the Foley catheter with 60 cc of sterile water before it is removed. The irrigation fluid was then suctioned away. The comparison of anastomotic suture placement concerned the part of the RP after division of the dorsal venous complex. After the initial placement of the anastomotic suture through the urethra, patients to were randomized to have a second bite taken deeply into the lateral pelvic fascia vs. no second bite.

Patients were randomized using the Clinical Research Database, a secure computer randomization system that ensures full allocation concealment. Blocks were of randomly selected length, stratified by treating surgeon (n = 6) and preoperative risk: low risk (PSA < 10 and biopsy Gleason ≤ 6), high risk (PSA > 20 or biopsy Gleason ≥ 8 or clinical stage ≥ T2c) or intermediate risk (not high or low risk). Patients were informed of their treatment allocation if they requested it.

The primary endpoints were the proportion of patients accured and the accrual rate. The secondary objective was to determine surgeon compliance with allocation and outcomes recording. We choose a sample size of 180. Based on an expected accrual rate of 50- 65% of eligible patients, the 95% Poisson confidence interval around 180 events in a unit period is 155-208 and the 95% exact binomial confidence interval for a proportion of 180 patients (e.g. data completion) is at most ± 8%.

In addition, we summarized the proportion of patients with eligible post-operative functional assessments, collected as part of routine clinical practice. Patients were defined as functional if they did not use routinely need continence pads in everyday life. We converted the time-to-event data into binary variables for the outcomes of 6 month and 12 month function. For 6 (12) month outcomes, patients who regained function before 7 (14) months after surgery were considered to have regained function; patients who were not functional after 5 (10) months and who did not regain function between 5 and 7 (10 and 14) months were considered non-functional. All analyses were conducted using Stata 11.0 (Stata Corp., College Station, Tx).

## Results

**Figure 1:**
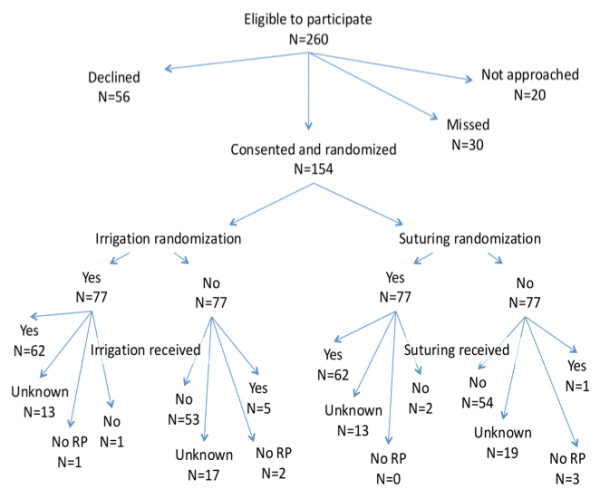
**Summary of patient consent and randomization**.

The trial was stopped shortly before achieving accrual goals when the Urology Service at Memorial Sloan-Kettering Cancer Center placed a temporary moratorium on all clinical trial consents due to staffing changes. Out of a total of 260 eligible patients, 154 (59%; 95% C.I. 53%, 65%) consented. Of the remainder, 56 patients declined to participate and 20 were not approached on recommendation of the treating surgeon. Thirty patients were not approached for logistical reasons, such as staff availability or patient time constraints (Figure 1).

As individual clinics were added to the study over time, we evaluated the accrual rate using the last 3 months of the study (January - March 2010), when all clinics were actively consenting patients. A total of 111 eligible patients were seen during this time, of which 67 were consented for a consent rate of 60%. Based on these numbers, we expect to accrue approximately 270 patients per year (95% CI: 208, 340). If we further assume that, with further experience of trial management and liberalization of the eligibility criteria - if a future study did not exclude patients for prior treatment - an accrual rate of 300 per year would seem a reasonable target.

There was wide variation in the consent and accrual rate across clinics. We piloted the trial in seven clinics. In the last three months of the trial, the consent rates across clinics ranged from 33% to 95% and the number of consents from 0 (a clinic where no eligible patients presented) to 21.

Table 1 summarizes the baseline characteristics of included patients. The cohort is typical of the trend towards treatment of higher risk patients following the increasing reliance on active surveillance at academic centers.

**Table 1:** Baseline characteristics of the study sample.

	No fascial suturing (n = 77)	Fascial suturing (n = 77)
	
Age	61.1 (57.8, 64.1)	61.3 (57.7, 65.3)
PSA at diagnosis	5.19 (3.66, 6.59)	5.17 (3.76, 6.59)

Clinical stage T1	57 (74%)	52 (68%)

Clinical stage T2a	12 (16%)	14 (18%)

Clinical stage T2b+	8 (10%)	11 (14%)

Biopsy Gleason grade		

6	28 (36%)	29 (38%)

7	42 (55%)	40 (52%)

8	7 (9%)	8 (10%)

Patients undergoing surgery	n = 74	n = 77

Pathologic Gleason grade^1^		

6	10 (14%)	8 (11%)

7	57 (77%)	59 (79%)

8	7 (9%)	8 (11%)

Positive surgical margins^2^	11 (15%)	9 (12%)

Seminal vesicle invasion	5 (7%)	7 (9%)

Extraprostatic extension	35 (47%)	36 (48%)

Lymph node invasion	6 (8%)	6 (8%)

	Irrigation (n = 77)	No irrigation (n = 77)
	
Age	60.3 (55.9, 65.2)	59.9 (56.1, 64.2)

PSA at diagnosis	4.95 (3.72, 6.90)	4.95 (3.66, 6.90)

Clinical stage T1	44 (57%)	49 (64%)

Clinical stage T2a	18 (23%)	16 (21%)

Clinical stage T2b+	15 (19%)	12 (16%)

Biopsy Gleason grade		

6	30 (39%)	29 (38%)

7	32 (42%)	34 (44%)

8	15 (19%)	14 (18%)

Patients undergoing surgery	n = 74	n = 76

Pathologic Gleason grade^1^		

6	9 (12%)	11 (15%)

7	59 (78%)	57 (76%)

8	8 (11%)	7 (9%)

Positive surgical margins^2^	11 (14%)	13 (17%)

Seminal vesicle invasion	10 (13%)	8 (11%)

Extraprostatic extension	35 (45%)	34 (45%)

Lymph node invasion	7 (9%)	7 (9%)

All values are median (IQR) or frequency (proportion).

1. Grade not assess in one patient due to neoadjuvant therapy

2. One patient missing data

Of the 154 patients randomized, 151 received radical prostatectomy. Figure 1 summarizes the number of patients who were treated according to their randomization. A non-trivial number of patients, around 1 in 5, were missing data on the actual treatment received. Nonetheless, in patients for whom treatment was recorded, this was inconsistent with allocation for only 6% (95% C.I. 2%, 10%), suggesting good surgeon compliance.

We explored predictors of protocol deviations, either in terms of failure to document procedures or differences between randomized and received procedure. There were no statistically significant associations with any patient characteristics (stage, grade, PSA or age) or treatment allocation (relative risk for irrigation of 0.64; 95% C.I. 0.37, 1.10; p = 0.12; relative risk for fascial suturing of 0.78; 95% C.I. 0.45, 1.40; p = 0.4). There were, however, clear differences between surgeons (p = 0.021). One surgeon had clearly lower documentation rates (61% vs. 79%); a different surgeon was responsible for 4 out of the 7 cases where treatment was inconsistent with allocation.

Table 2 summarizes the number of patients with currently available functional outcome data. An acceptable proportion of patients had eligible assessments at 6 (65%; 95% C.I. 57%, 72%) and 12 months (71%; 95% C.I. 63%, 78%). Furthermore, we expect some of the patients currently with no assessments or a last assessment before the eligibility window to return for an assessment in the future, potentially increasing the number of patients with eligible functional outcome data. We did not find statistically significant associations between missing data and any patient characteristics or treatment allocation (relative risk for irrigation 0.99; 95% C.I. 0.60, 1.60; p = 1; relative risk for suturing 0.88; 95% C.I. 0.53, 1.40; p = 0.6). However, again there were marked differences between surgeons (p = 0.034). The total number of patients with missing documentation of procedure or missing outcome data was 65 (43%; 95% C.I. 36%, 51%).

**Table 2:** Summary of functional outcomes assessment for patients on protocol 09-051 who received radical prostatectomy (n = 151).

	Continent	
	**6 months**	**12 months**

Yes	68	99

No	30	8

First assessment after window* & functional	2	0

Last assessment before window* & not functional	38	31

*No assessments*	13	

*window is defined as 10-14 months for 12 month assessment and 5-7 months for 6 month assessment.

Although we did not expect to find important differences between groups in this underpowered feasibility study, we did conduct exploratory analyses of outcome by group. A total of 13 patients experienced a biochemical recurrence, 8 among those randomized to receive urethral irrigation and 5 among those randomized not to receive the intervention (hazard ratio 1.6; 95% C.I. 0.51, 4.81). Continence at one year was reported by 54 out of 56 patients randomized to fascial involvement in the placing of the anastomotic sutures (96%) compared to 45 out of 51 in patients randomized to have no fascial suturing (88%; risk difference 8%, 95% C.I., -2%, 18%).

## Discussion

We have demonstrated the feasibility of a clinically-integrated randomized trial. First, the trial was conducted at extremely low cost. With the exception of protocol writing and data analysis, and start-up meetings with surgeons and on-going trial monitoring, the only non-trivial expenditure of time or money was for consenting patients. Clinic staff could readily identify and flag eligible patients with a negligible expenditure of effort. We found that surgeons could usually explain the idea behind the trial to patients in 2 - 3 minutes. Consent paperwork was handled by research assistants concurrent with other standard consents, such as for tissue use protocols. The process of randomization - stratification, faxing of consent documents, and communication of results to surgical fellows - took research assistants 5 - 10 minutes per patient. Trial data were directly downloaded from the clinical database by the trial statistician. In comparison with a traditional trial, our study avoided many costs - including those for research assistants to follow patients over time as well as the cost of data abstraction and data entry - but did not incur any additional costs, as all aspects of the clinically-integrated trial (e.g. consent, start up meetings) are also a necessary part of more traditional designs.

Second, randomization did indeed become a routine part of clinical practice, with approximately 80% of patients being approached and close to 75% of those agreeing to participate. Third, we were able to obtain outcome data on a high proportion of patients, despite there being no attempt whatsoever to follow trial patients differently outside of routine clinical care.

Various lessons were learned during study start up and implementation. Before the first patient was randomized, trial staff spent time with clinic patients discussing how different ways of describing the trial would affect the degree of comfort that they would feel in participating. The major concern of patients concerned whether they might receive substandard care if they took part. Patients appeared particularly averse to descriptions of the trial suggesting that treatment would be given at random. Accordingly, in both the written consent form (see additional file 2) and in oral presentations of the trial, the "uncertainty principle"[12] was stressed. Patients were told that surgeons would always use their clinical judgment, and would choose the treatment approach that would lead to the best outcome; if and only if the surgeon was genuinely unsure of which approach to take would the randomized allocation be accessed. Discussions with patients also revealed that it was critical that the trial first be introduced to them by their surgeon; being approached by a research assistant or even clinical fellow (a surgeon in training working with the attending surgeon) would lead patients to suspect that their surgeon was not fully confident about the trial.

Given the critical role that administrative clinic staff played in identifying eligible patients and bringing them to the attention of surgeons, we expended considerable effort involving clinical staff in trial start up (see additional file 3). As each surgeon ran clinics in a slightly different way, we relied on clinic staff to suggest trial procedures. For example, one clinic nurse suggested including a brightly colored reminder notice in the case file given to the surgeon before entering the consultation room. Involving staff in this way increased "ownership" in the trial and provided incentives for high accrual rates. To complement this approach, we tracked consent rates for different clinics, bringing the results to the attention of surgeons and their clinic staff.

Surgical fellows play a key role in the everyday running of clinics, in most instances, being the first doctor who sees a patient considering surgery. Working closely with the surgical fellows therefore also became a key aspect of efficient trial management. Indeed, we often saw large changes in accrual rates within a particular clinic as fellows rotated. We were keen to emphasize the importance and novelty of the trial and to appeal to fellows' commitment to evidence-based medicine.

That said, encouragement of surgeons, surgical fellows and non-medical clinic staff may have been unproductive without the full support of the surgical leadership of the hospital. The co-principal investigator was the chair of the Department of Surgery. It is difficult to imagine that the trial would have accrued without this enthusiastic endorsement.

Nonetheless, further attention to routine systems of data gathering will be required before the methodology can be optimized. Recording of compliance with randomization was missing in about 20% of cases, and so clearly additional procedures need to be established to ensure this key aspect of documentation. In particular, we propose adding easy to use "tick boxes" in the operative record. Doing so would not only ease documentation, but would allow the study team to conduct on-going monitoring of compliance both with documentation and with treatment allocation. This would allow identification of surgeons with poor compliance and suitable intervention.

Recording of patient outcome, while adequate, was also less than perfect. Since the protocol was opened, we have moved to entirely electronic reporting of patient outcomes, via emails to patients at home or iPads in the clinic. To assess how this new system affects patient reporting, we studied all patients treated by radical prostatectomy between January 2010 (towards the end of the trial, when the electronic recording was fully implemented) and October 2010 (to allow all patients to have 14 months of follow-up). During this period, 599 patients were treated and we obtained data for urinary function at one year from 498, a data completion rate of 83%. We are also in the process of implementing a system that provides feedback to patients on the basis of their answers, for example, recommending referral to a voiding dysfunction specialist to patients who report urinary dysfunction [13]. We anticipate that improving use of patient-reported outcomes in clinical practice - an approach that has been shown to improve doctor-patient communication [14] and decrease symptom intensity [15] - will also increase data completion rates in subsequent clinically-integrated trials. We also recommend the use of sensitivity analysis in any subsequent, fully powered trial, to determine whether missing data may have influenced the strength or direction of results.

That said, we are confident that the rates of data completion we report here - even if suboptimal - fully justifies the clinically-integrated randomized trial methodology. With respect to missing documentation on surgical approaches, we have no reason to believe that missing data reflected treatment choices: in discussion with clinicians, it seemed that documentation failures were inadvertent. Naturally, we cannot entirely rule out bias with respect to documentation. This is partly a function of having relatively wide confidence intervals around the estimates of differences between groups. Perhaps more importantly, the possibility of bias may change depending on the surgeons and comparison involved. For example, it might be that in some other implementation of the clinically-integrated trial methodology, a sub-group of surgeons with strong preferences might attempt to subvert the trial by selective documentation. As such, careful monitoring of documentation rates, and statistical comparisons of patients with and without documentation of the procedure used, will be important in any clinically-randomized trial.

With respect to missing outcome data, we saw no evidence that this varied by patient characteristics. To determine whether our 29% rate of missing data is in anyway extreme or outlying in the context of randomized trials in general, we examined typical rates in other fields. In two studies examining reports in major medical journals, about 20% of trials had a rate of missing data more than 20%[16,17]. However, rates do vary depending on the patient group and length of follow-up: mean 30% at one year in weight loss research [18]; mean 37% for short-term studies of depression [19]; 20% of rheumatology trials had more than 30% missing data [20]. It is of note that in each of these research areas, the likelihood of bias due to missing data is far higher than for the current trial. There are obvious reasons why drop-out would be associated with inefficacy in depression or weight-loss trials and with medication side-effects in rheumatology trials. In contrast, it is hard to see how a patient's allocation or continence status would affect his propensity to continue with clinical follow-up. In contrast, patients return for follow-up after radical prostatectomy to check for recurrence. If a patient with urinary dysfunction was more or less likely to return for a cancer check, then the mechanism for this is far less obvious than how continuing depression would affect a patient's willingness to continue on a drug study.

As such, missing outcome data is largely an issue of decreased sample size. A more traditional approach to the randomized trial, where patients would complete protocol-specific questionnaires under close monitoring by study staff, might well have a higher overall rate of data completion. Given the expense of such trials, and the lowered patient acceptance of and recruitment to studies that involve additional reporting burden, the overall number of patients providing data would likely be higher with a clinically-integrated trial approach. This might also be explained in a "value of information" context [21]: the cost per data point is dramatically lower for the clinically-integrated trial, so given a fixed research budget, this approach will result in more information to help guide clinical practice.

## Conclusions

We have demonstrated the feasibility of a clinically-integrated randomized trial, an approach that allows trials to be run at very low cost, with minimal disruption to patients and clinicians. A fully powered version of a radical prostatectomy trial is now underway. Due to incomplete documentation of surgical approach we report here, the follow-up trial includes specific procedures to aid documentation, by incorporating "tick boxes" in the surgical medical record. We encourage other researchers to consider how our methodology might be applied to different research questions.

## List of abbreviations

CI: Confidence interval; MSKCC: Memorial Sloan Kettering Cancer Center; PSA: Prostate specific-antigen; RP: Radical prostatectomy

## Competing interests

The authors declare that they have no competing interests.

## Authors' contributions

The overall methodology was conceived by AJV, with input from PTS. The design of the protocol was by AJV and PTS, with input from all authors. CB was responsible for the statistical analysis. AJV and CB drafted the manuscript, the final version of which was read and approved by all authors.

## Supplementary Material

Additional file 1 **Appendix A 09 051 write up description of surgical techniques**. Detailed description, including diagrams, of the surgical techniques compared in the trial. Click here for file

Additional file 2 **Appendix B 09 051 write up informed consent**. The informed consent used in the trial. Click here for file

Additional file 3 **Appendix C 09 051 write up brief for clinical staff**. A briefing document circulated to clinical staff in urology that describes the trial. Click here for file
