# Supplementary material for: Feasibility study of a clinically-integrated randomized trial of modifications to radical prostatectomy
Source: Trials. 2012 Feb 24;13:23. doi: 10.1186/1745-6215-13-23 (PMC3298715; doi:10.1186/1745-6215-13-23)
Supplement: Additional file 1 — Appendix A 09 051 write up description of surgical techniques. Detailed description, including diagrams, of the surgical techniques compared in the trial. [file 1745-6215-13-23-S1.PDF]

APPENDIX: DETAILED DESCRIPTION OF THE INTERVENTIONS

Two aspects of the operation may vary, fascial suturing and urethral irrigation. For each aspect, surgeons will use their clinical judgment as to the best interests of the patient. In other words, if there are clear reasons to use or avoid a fascial suturing approach, the surgeon will act accordingly; similarly, if there is a clear reason to irrigate or avoid irrigating the urethra, the surgeon can make the appropriate clinical decision. If the surgeon is unsure as to which approach to take, then the randomization scheme will be followed.

1. Fascial suturing

For patients randomized to both fascial suturing and no fascial suturing: after division of the dorsal venous complex and control of bleeding, the levator ani fibers are dissected away from the apex of the prostate exposing the urethra. The operative field should be dry enough to permit precise division of the anterior urethra and placement of the anastomotic sutures under direct vision (Figure 1). To secure the cut edge of the urethra we place the anastomotic sutures at this point, rather than attempt to place sutures in the retracted urethral stump later in the procedure. Four 00 Monocryl absorbable sutures on a UR-6 needle are placed from inside out just 3 mm into the urethra.

For patients undergoing fascial suturing only, after the initial placement of the suture through the urethra a second bite is taken deeply into the fascia of the lateral pelvic fascia (Figure 1, panel C). For patients in both groups, Next, the catheter is withdrawn, exposing the undivided posterior urethra and the firm fibrous layer of Denonvilliers' fascia beneath (Figure 2). Two additional posterior sutures are placed through the posterior layer of fascia and through the urethra, from outside in, at the 5 and 7 o'clock positions. These two sutures must be well away from the previously mobilized neurovascular bundles. Finally, the posterior urethra and rectourethralis muscle are divided and the prostate is dissected away from the rectum beneath Denonvilliers' fascia (Figure 2).

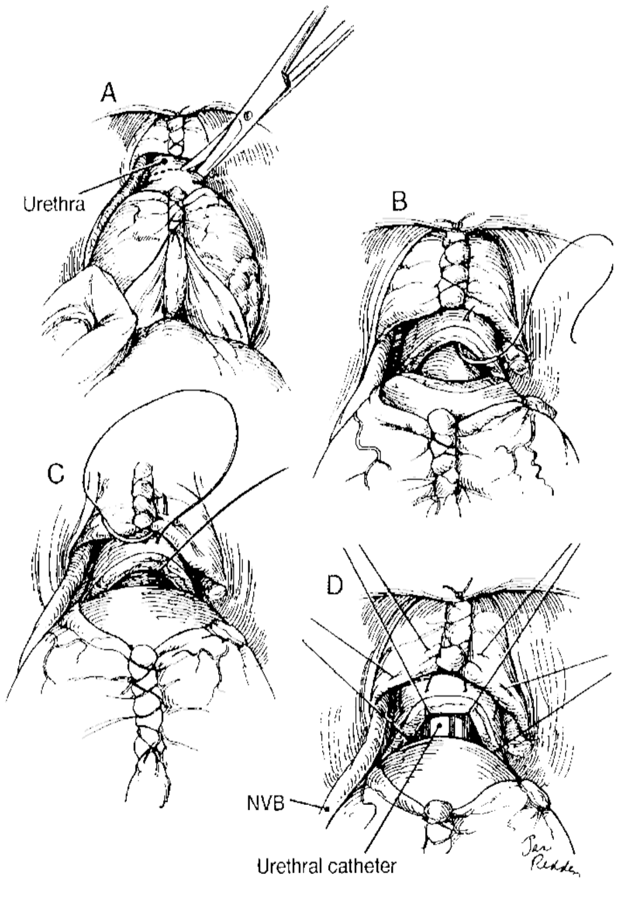

FIGURE 1. Close-up views of urethra at the prostatic apex, illustrating the site of anterior division **(A,B)** and the placement of the anterior anastomotic sutures beneath the mucosa of the urethra and then separately through the thick layer of lateral pelvic fascia **(C,D)** that was oversewn to control the dorsal vein complex. (NVB, neurovascular bundle)

FIGURE 2. After the nerves have been dissected free (or divided), the remaining urethra and posterior layer of Denonvilliers' fascia beneath it are divided (A). Two posterior anastomotic sutures are placed at 5 and 7 o'clock through the fascia and urethra (A). The correct plane of dissection adjacent to the rectum is determined with the aid of a Kitner dissector (B). (NVB, neurovascular bundle.)

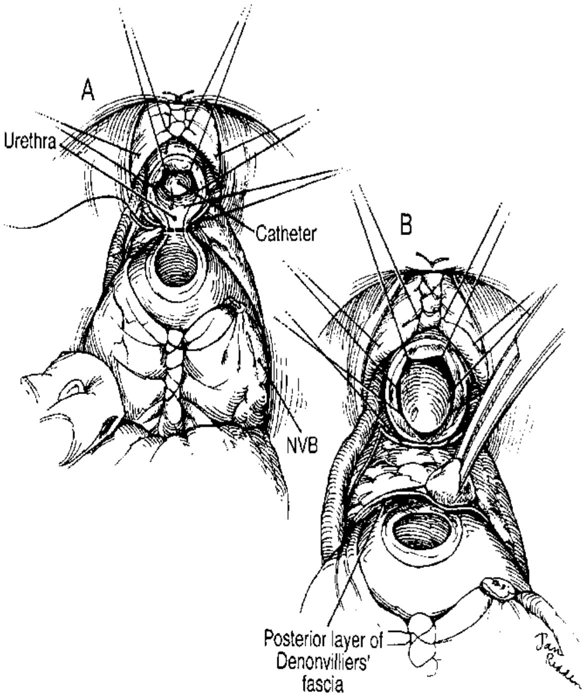

2. Irrigation

The technique is summarized in Figure 3. The anterior two-thirds of the urethra is divided exposing a Foley catheter that was placed at the beginning of the procedure. Four anterior anastomotic sutures are placed through the cut-edge of the membranous urethra. The Foley catheter will then either be simply withdrawn from the urethra and removed or irrigated with 60 cc of sterile water as it is withdrawn and removed with the irrigation fluid suctioned.

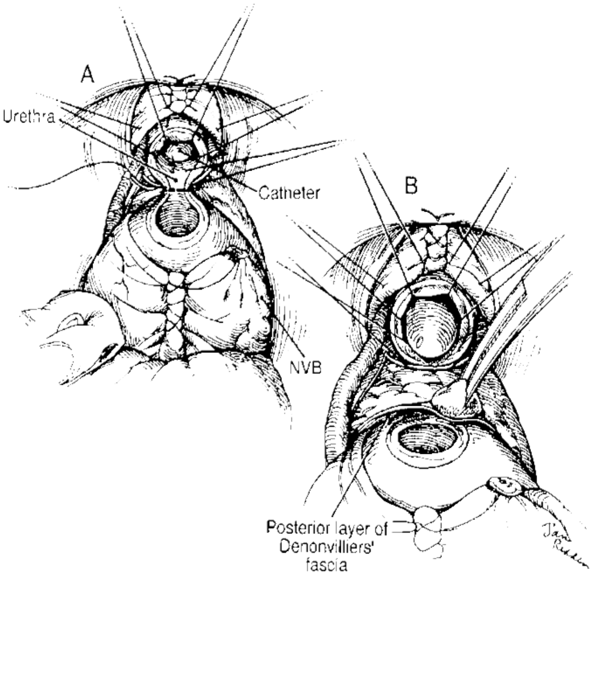

FIGURE 3. Close-up views of urethra at the prostatic apex, illustrating the site of anterior division of the urethra. Following placement of the four anterior anastomotic sutures, the Foley catheter will either be simply withdrawn from the urethra or irrigated with 60 cc of sterile water as it is withdrawn from the patient to 'wash' the urethra.
