# Supplementary material for: Feasibility study of a clinically-integrated randomized trial of modifications to radical prostatectomy
Source: Trials. 2012 Feb 24;13:23. doi: 10.1186/1745-6215-13-23 (PMC3298715; doi:10.1186/1745-6215-13-23)
Supplement: Additional file 2 — Appendix B 09 051 write up informed consent. The informed consent used in the trial. [file 1745-6215-13-23-S2.PDF]

PATIENT INFORMED CONSENT FOR CLINICAL RESEARCH

A Randomized Trial Of Modifications To Radical Prostatectomy: Feasibility Study

You have been asked to participate in a research study. In order to decide whether or not you should agree to be part of this research study, you should know enough about its risks and benefits in order to make a sound judgment. This process is known as informed consent.

This is a clinical trial, a type of research study. Your study doctor will explain the clinical trial to you. Clinical trials include only people who choose to take part. Please take your time to make your decision about taking part. You may discuss your decision with your friends and family. You can also discuss it with your healthcare team. If you have any questions, you can ask your study doctor for more explanation.

This consent form gives you detailed information about the research study. Once you understand the study, its risks, and its benefits, you will be asked to sign the form if you wish to take part. You will be given a copy to keep.

You are being asked to take part in this study because you have prostate cancer that will be treated by surgery ("radical prostatectomy").

Why is this study being done?

This study aims to determine whether surgeons at Memorial Sloan-Kettering Cancer Center are able to randomize patients to test modifications of surgery to remove the prostate. Surgery to remove the prostate is known as a "radical prostatectomy". Surgeons know many things about the best way to do a radical prostatectomy. However, there is disagreement about some aspects of surgery. Two modifications of surgery to remove the prostate (radical prostatectomy) identified for this study include Irrigation, and Fascial Suturing.

Irrigation. Cancer cells can spill during surgery and this can cause cancer to return ("recur"). Some surgeons believe that "irrigating" could help stop spilling of cancer cells. "Irrigating" means washing the surgical area with sterile water and sucking the water back up through a tube. As a result, surgeons vary as to how they irrigate. In this study, we will examine irrigation of the urethra. This is the part of the body that carries urine from the bladder to the penis.

Fascial suturing. Surgeons believe that what happens to the urethra can affect the risk of incontinence. This is when a patient cannot control urine, and drips or leaks urine. One idea is that additional stitches ("sutures") to the connective tissue ("fascia") could be helpful, but this is not known for sure.

All of the surgeons who are taking part in this study have used these techniques at different times. However, they are unsure as to the best approach. Sometimes, they use different treatments with

different patients. In total, 180 subjects will be evaluated to determine the feasibility of recruitment at Memorial Sloan Kettering.

Is there a potential conflict of interest for this study?

There are no known investigator and/or institutional conflicts of interest for this study.

How many people will take part in the study?

About 180 people will take part in this study at Memorial Sloan-Kettering Cancer Center. Your surgeon is one of 8 surgeons who are recruiting patients for this study.

What will happen if I take part in this research study?

Before you begin the study …

All patients having surgery for prostate cancer undergo a physical examination and a blood test. They also complete a quality of life questionnaire. Some patients are referred for a scan ("MRI"). These standard clinical evaluations would occur whether or not you decide to take part. There are no extra tests you would be asked to do.

During surgery …

Your surgeon will treat your prostate cancer with standard surgery methods. Two aspects of the surgery may vary between different patients on the trial, urethral irrigation and fascial suturing. As described above, these are: fascial suturing and urethral irrigation. The approach you receive will depend on your surgeon's best judgment as what would be most effective for you. However, it may be that, during the operation, your surgeon sees no clear reason to treat one way or the other. If in your surgeon's judgment it is a 50:50 decision, then the approach you receive will be determined at random by a computer. You will receive one of four different treatments, with an equal chance of each:

- urethral irrigation but no fascial suturing
- fascial suturing but no urethral irrigation
- both urethral irrigation and fascial suturing
- neither urethral irrigation nor fascial suturing

You can find out which treatment you received by asking your surgeon.

After my surgery …

There are no additional tests, procedures or exams for this trial. As part of routine care, you will be asked to return for blood tests. The blood test is to see whether the cancer has returned. You will also be asked to complete questionnaires. These will ask how surgery has affected your sexual and urinary function. The information from these questionnaires is entered into your case notes to help your surgeon manage your care.

How long will I be in the study?

All patients undergoing radical prostatectomy at Memorial Sloan-Kettering Cancer Center are followed carefully for many years. If you take part in this study, you will receive the same follow-up as any other patient treated by radical prostatectomy.

Can I stop being in the study?

Yes. You can decide to stop at any time. Tell the study doctor if you are thinking about stopping or decide to stop. The study doctor may stop you from taking part in this study at any time if he/she believes it is in your best interest or if the study is stopped.

What side effects or risks can I expect from being in the study?

Surgery for prostate cancer is associated with various risks.

- All patients are unable to have an erection after surgery. Many, but not all, men then recover the ability to have an erection within 1 – 2 years.
- All patients initially have problems with urination, but most then slowly recover over time. However, some patients have problems with urination for many years.
- In some cases, surgery leads to pain when passing urine or stools.
- Serious problems after surgery, such as a heart attack, are very rare.

Your surgeon will discuss the side effects and risks of surgery with you. You will also get further information when you sign the consent for your surgery. All of the surgical techniques used in the study are routinely used by surgeons in their clinical care. As a result, there are no risks or side-effects specifically due to being on the study.

Are there benefits to taking part in the study?

Taking part in this study may or may not make your health better. We do know that the information from this study will help doctors learn more about surgery for prostate cancer. This information could help future cancer patients.

What other choices do I have if I do not take part in this study?

If you do not take part in the study you will receive a surgery from your surgeon as normal. Your surgeon would explain the details of the surgery they would do since each surgeon may use a different approach.

Will my medical information be kept private?

Every effort will be made to keep your study records private. It is the responsibility of the research staff at Memorial Hospital to make sure that your records are managed to protect your privacy. If information from this study is used in any reports or publications, your name and anything else that could identify you will not be used. Trained staff at Memorial Hospital may review your records if necessary. Access to your medical information will be limited to those listed in the Research Authorization Form, which is a part of the informed consent process.

What are the costs of taking part in this study?

You and/or your health plan/ insurance company will need to pay for some or all of the costs of treating your cancer in this study. Some health plans will not pay these costs for people taking part in studies. Taking part in this study will not cost your insurance company more than the cost of getting regular cancer treatment.

You will not be paid for taking part in this study.

What happens if I am injured because I took part in this study?

You will get medical treatment if you are injured as a result of taking part in this study. You and/or your health plan will be charged for this treatment. The study will not pay for medical treatment.

What are my rights if I take part in this study?

Taking part in this study is your choice. You may choose either to take part or not to take part in the study. If you decide to take part in this study, you may leave the study at any time. No matter what decision you make, there will be no penalty to you and you will not lose any of your regular benefits. Leaving the study will not affect your medical care. You can still get your medical care from our institution.

We will tell you about new information or changes in the study that may affect your health or your willingness to continue in the study.

In the case of injury resulting from this study, you do not lose any of your legal rights to seek payment by signing this form.

Who can answer my questions about the study?

You can talk to your surgeon about any questions or concerns you have about this study. If you would like to speak to a researcher about the study, contact Andrew Vickers, Ph.D. at (646) 735 8142.

Any hospital that does research on people has an institutional review board (IRB). This board reviews all new studies to make sure that the patient's rights and welfare are protected. The IRB at MSKCC has reviewed this study.

For a non-physician whom you may call for more information about the consent process, research patients' rights, or research related injury is Jorge Capote, RN, Patient Representative, telephone number: (212) 639-8254.

PATIENT INFORMED CONSENT FOR CLINICAL RESEARCH
A Randomized Trial Of Modifications To Radical Prostatectomy: Feasibility Study

Statement of professional obtaining consent

I have fully explained this research study to the research participant or guardian of research participant ____________________. In my judgment and the research participant's, there was sufficient access to information, including risks and benefits to make an informed decision.

Date: ________ Consenting Professional Signature: ____________________________

Consenting Professional Name:__________________________________
(Print)

Research Participant (or guardian's) statement

I have read the description of the clinical research study or have had it translated into a language I understand. I have also talked it over with the consenting professional to my satisfaction. I understand that my/the research participant's participation is voluntary. I know enough about the purpose, methods, risks, and benefits of the research study to judge that I want (the research participant) to take part in it.

Research Participant number: _______Research Participant Signature:_____________________

Date: ______________________ Research Participant Name: ______________________
(Print)

IRB PB

RESEARCH AUTHORIZATION

A Randomized Trial Of Modifications To Radical Prostatectomy: Feasibility Study

Research Participant Name: ____________________________

Research Participant MRN :_____________________________

We understand that information about you and your health is personal. We are committed to protecting the privacy of your information. Because of this commitment, we must obtain approval from you before we can use your protected health information for research purposes. This form provides that authorization. This form also helps us make sure that you are informed of how this information will be used or disclosed in the future. Please read the information below carefully before signing this form.

USE AND DISCLOSURE COVERED BY THIS AUTHORIZATION

A representative of Memorial Sloan-Kettering Cancer Center must answer these questions completely before providing this authorization form to you. *PLEASE DO NOT SIGN A BLANK FORM*. ***You or your personal representative should read the descriptions below before signing this form.***

Who will have access to and/or use your health information?

The following individuals and/or organization(s) may have access to use, disclose or receive some information about you. They may only share the information to the individuals/parties indicated on this list. This information must be shared with you, the research subject and/or your personal representative, as required by law.

- ☑ Every research site for this study, including Memorial Sloan-Kettering Cancer Center and the research support staff (for example, research study assistant) and medical staff at each location
- ☑ Every health care personnel who provides services to you in connection with this study
- ☑ Any laboratories, other individuals/organizations that analyze your health information in connection with this study as defined by protocol
- ☑ The following research sponsors: Memorial Sloan-Kettering Cancer Center
- ☑ The National Cancer Institute and/or the National Institute of Health
- ☑ The United States Food and Drug Administration and other regulatory agencies responsible for oversight.
- ☑ The members and staff of the hospital's Institutional Review Board and Privacy Board
- ☑ Principal Investigator and Co-Principal Investigator(s): Andrew Vickers, Ph.D., and Peter Scardino, M.D.
- ☑ Members of the Research Team including the participating investigators, research assistants, clinical nurses, fellows/residents, and clerical support staff.

- ☑ Members and staff of the hospital's Office of Clinical Research, Computing Resource Group that manages research databases, and the research management and support staff in the clinical departments
- ☑ Members of the Hospital's Data Safety Monitoring Board/Committee and Quality Assurance Committee

<u>What information will be used or disclosed?</u>

The boxes checked below should provide you with enough detail so that you can understand what information may be used or disclosed.

- ☒ Your entire research record
- ☒ Any part of your medical records held by the hospital

- ☒ HIV-related information. This includes any information indicating that you have had an HIV-related test, or have HIV infection, HIV-related illness or AIDS, or any information which could indicate that you have been potentially exposed to HIV. (New York State requires us to obtain special consent)

SPECIFIC UNDERSTANDINGS

By signing this form, you give permission for the sharing of your protected health information noted above. The purpose for the use and disclosure of your information, is to conduct the research study explained to you during the informed consent process. This form also ensures that the information relating to the research is available to everyone who may need it. Your protected health information may also be used for your research treatment, to collect payment for your treatment while on the study (when applicable), and to run the business operations of the hospital.

Once we have shared your information with the individuals and organizations listed on this form, they may be able to share your information again, if they are not subject to laws that protect your privacy.

It is your right to refuse to sign this authorization form. If you do not sign this form, you will not be able to participate in the research study. You will not receive the research treatment that was described to you. Your health care outside the study will not be affected. The payment for your health care or your health care benefits will not be affected.

If you sign this authorization form, you will have the right to withdraw it at any time. To withdraw the authorization will prohibit further use or disclosure of your health information. If the hospital has already use your health information approved by your authorization or needs the information to fulfill an obligation or analyze the data, the use or disclosure can not be stopped. This authorization form will not expire unless you withdraw it. If you want to withdraw this authorization, please write to Andrew Vickers, PhD at the hospital.

You have a right to see and copy your health information described in this authorization form in accordance with the hospital's policies. You also have a right to receive a copy of this form after you have signed it.

<u>Notice Concerning HIV-Related Information</u>

If you are authorizing the release of HIV-related information, you should be aware that the individuals/organizations are prohibited from sharing any HIV-related information without your approval unless permitted to do so under federal or state law. You also have a right to request a list of people who may receive or use your HIV-related information without authorization. If you experience discrimination because of the release or disclosure of HIV-related information, you may contact the New York State Division of Human Rights at (800) 523-2437 or (212) 480-2493 or the New York City Commission of Human Rights at (212) 306-7450 or (212) 306-7500. These agencies are responsible for protecting your rights.

SIGNATURE

I have read this form and all of my questions have been answered. By signing below, I acknowledge that I have read and accept all of the information above.

____
Signature of Research Participant or Personal Representative

____
Print Name of Research Participant or Personal Representative

____
Date

____
Description of Personal Representative's Authority
