# Supplementary material for: Feasibility study of a clinically-integrated randomized trial of modifications to radical prostatectomy
Source: Trials. 2012 Feb 24;13:23. doi: 10.1186/1745-6215-13-23 (PMC3298715; doi:10.1186/1745-6215-13-23)
Supplement: Additional file 3 — Appendix C 09 051 write up brief for clinical staff. A briefing document circulated to clinical staff in urology that describes the trial. [file 1745-6215-13-23-S3.PDF]

General comments

Some key phrases to use with patients:

- We are a research hospital
- We are always trying to fine tune our techniques
- Dr [surgeon name] includes his patients in research as a matter of routine
- Dr [surgeon name] will always use his surgical judgment at every point of your surgery. It is only if he is unsure what to do that he will let the computer decide

Patient New Visit

Surgeons should try to raise the general issue of the study at each patient's new visit. What you should say is something along the following lines:

> As you probably know, Memorial Sloan-Kettering Cancer Center is a research hospital, and one of the reasons that we are able to give the very best treatments is that we are always conducting research on what works best. I just wanted to introduce briefly some research we are doing; we can talk about this in more detail should you decide to have surgery.
>
> Now as I am sure you can imagine, the radical prostatectomy is a very complicated operation and there are literally hundreds of different steps involved. We know pretty much exactly how to do most of these steps. However, there are a few we are a little unsure of. We are always trying to fine tune our techniques.
>
> We have some research on radical prostatectomy going on and I try to involve all my patients as a matter of routine. In brief, I will do my normal operation in the normal way. But there are two particular steps where there are two different ways of doing the step. What I'll do is have a look at the situation, and if it looks as though I should do things one way or the other, then that is what I will do. However, if I am unsure – if in my judgment as a surgeon there is no good reason to use one or other method – then I will let a computer decide at random. Other than that, everything is the same: there are no extra tests, questionnaires or visits for patients on the trial

If a new visit goes directly to consent, the nurse and / or RSA should check with the surgeon whether they have discussed the trial with the patient. The fellow should then provide more details about the trial, something along the following lines:

> I understand that Dr [surgeon name] has told you about some of the research that we are doing here. I wanted to tell you a little bit more about our trial that aims to fine tune some of the techniques that we use during radical prostatectomy. There are two things we are looking at.
>
> The first concerns how we manage the urethra, the part of your body that carries urine from the bladder to the penis. The urethra goes through the middle of the

prostate, so we have to cut it when we remove the prostate. There is a disagreement as to how to sew the urethra back up: some surgeons sew it just to the bone; some to the bone and to soft tissue; some surgeons sometimes do it one way and sometimes do it the other.

The second thing that we are looking at concerns the catheter that is placed into your bladder. This has to be removed during surgery. Some surgeons think that it might be important to wash this out very thoroughly to prevent any cancer cells leaking into the body; other surgeons think that, if anything, washing the catheter would make it more likely for cancer cells to leak.

Now if you take part in the trial, pretty much everything will happen as normal: you don't have to take any additional tests or fill in additional forms, and your surgery will be the normal surgery of Dr [surgeon name]. However, when Dr [surgeon name] comes to sew up the urethra, he will look to see if there is any reason to sew it just to the bone or whether he really needs to include the soft tissue as well. This might depend, for example, on your anatomy, or on how the operation is going. If Dr [surgeon name] sees a good reason to use soft tissue or just bone, then that is what he will do. If he is unsure, and does not see any reason to do things one way or the other, we will ask the computer, which will decide at random. Similarly, when it comes to removal of the catheter, Dr [surgeon name] will see if there is a good reason to do things one way or another, but consult the computer if he is unsure.

Consent visits

RSA to remind fellow, attending, and nurse that patient is eligible for the trial. The fellow introduces the trial using the script above.

Frequently asked questions:

1. **Don't we know which way is better?** There are a lot of things we know about the radical prostatectomy. For example, we know that nerve sparing surgery leads to better outcome in terms of impotence and incontinence and so we always try to do a nerve sparing operation. But there are some thing we don't know how to do best, and we are always trying to fine tune our knowledge. Now if we did know whether it was best to sew the urethra to the soft tissue or just the bone, none of the surgeons would take part in the trial and the hospital wouldn't allow us to do it: there is very careful review to ensure that all patients on studies get the very best care.
2. **I don't like the idea of a computer making a decision.** It will always be your surgeon making the decision as to what to do to make your surgery most effective. If it is only if he is unsure what to do – if there is no obvious reason to do things one way or another – that he consult the computer.
3. **How does Dr [surgeon name] normally do it?** Some surgeons at Memorial Sloan-Kettering Cancer Center use one technique, some use the other, and some

do it sometimes one way and other times another way. However, when we started the trial, all surgeons sat around the table and discussed various aspects of the surgery. There were obviously many things that the surgeons did that they felt worked best – or that there was good scientific evidence for – and these were not considered for study. For the two techniques were are studying in the trial – fascial suturing and irrigation – there was agreement amongst all surgeons that it wasn't clear what the best method was, and that we should conduct a trial to find out. So there won't be a "normal" way of doing the surgery until we find out what works best at the end of the trial.

4. **Is this some new "radical" treatment?** No, these treatments have both been used by surgeons for a long time and both have been shown to be safe.
